# Multibiomarker panels in liquid biopsy for early detection of pancreatic cancer – a comprehensive review

**DOI:** 10.1186/s13046-024-03166-w

**Published:** 2024-09-02

**Authors:** Kim-Lea Reese, Klaus Pantel, Daniel J. Smit

**Affiliations:** Institute of Tumor Biology, University Medical Center Hamburg-Eppendorf, Martinistraße 52, Hamburg, 20246 Germany

**Keywords:** Blood-based biomarkers, Early-stage diagnosis, Liquid biopsy, Multimarker panel, Pancreatic cancer

## Abstract

Pancreatic ductal adenocarcinoma (PDAC) is frequently detected in late stages, which leads to limited therapeutic options and a dismal overall survival rate. To date, no robust method for the detection of early-stage PDAC that can be used for targeted screening approaches is available. Liquid biopsy allows the minimally invasive collection of body fluids (typically peripheral blood) and the subsequent analysis of circulating tumor cells or tumor-associated molecules such as nucleic acids, proteins, or metabolites that may be useful for the early diagnosis of PDAC. Single biomarkers may lack sensitivity and/or specificity to reliably detect PDAC, while combinations of these circulating biomarkers in multimarker panels may improve the sensitivity and specificity of blood test-based diagnosis. In this narrative review, we present an overview of different liquid biopsy biomarkers for the early diagnosis of PDAC and discuss the validity of multimarker panels.

## Background

The most common type of pancreatic cancer is pancreatic ductal adenocarcinoma (PDAC), which accounts for more than 90% of all pancreatic cancers [1]. PDAC-related precancerous conditions include pancreatic intraepithelial neoplasms (PanINs), intraductal papillary mucinous neoplasms (IPMNs), and mucinous cystic neoplasms (MCNs). The etiology of pancreatic cancer is not fully understood, but several risk factors are associated with PDAC. In addition to common cancer risk factors, including age, obesity, genetic predispositions, smoking and alcohol consumption, the factors conferring the highest risk are type 2 diabetes mellitus and chronic pancreatitis [2].

Pancreatic cancer is the seventh most commonly diagnosed cancer and the fourth most frequent cause of cancer-related deaths in Europe; it accounted for almost as many diagnoses (140,116 cases) as deaths (132,134 deaths) in 2020 [3]. Despite tremendous efforts in research and new therapies resulting in increased survival rates of patients with other cancer types, pancreatic cancer still has a low 5-year survival rate of approximately 10%, with a median overall survival (OS) of less than six months [4]. One of the main reasons is late diagnosis, as patients do not show specific early clinical symptoms [5]. At the time of PDAC detection, less than 20% of tumors are eligible for curative resection [6]. However, surgery followed by adjuvant systemic chemotherapy is the best therapeutic option, significantly increasing the 5-year survival rate [7]. At advanced tumor stages with metastases, the only remaining treatment is systemic chemotherapy, which has a low response rate and a high resistance rate [6]. Consequently, it is highly important to develop diagnostic tests that enable the detection of early-stage PDAC (AJCC/UICC stages I and II) to improve the OS and progression-free survival (PFS) of patients.

PDAC is mainly diagnosed through medical imaging methods, including computer tomography, magnetic resonance imaging, magnetic or endoscopic retrograde cholangiopancreatography, and endoscopic ultrasound-guided fine needle aspiration. However, a clear diagnosis is not always possible because of the retroperitoneal location of the pancreas and the small size of early-stage PDAC lesions [6, 8]. In addition, screening for the early diagnosis of PDAC by imaging is not practical, as it is neither cost nor time efficient and involves exposure to radiation [8].

**Fig. 1:**
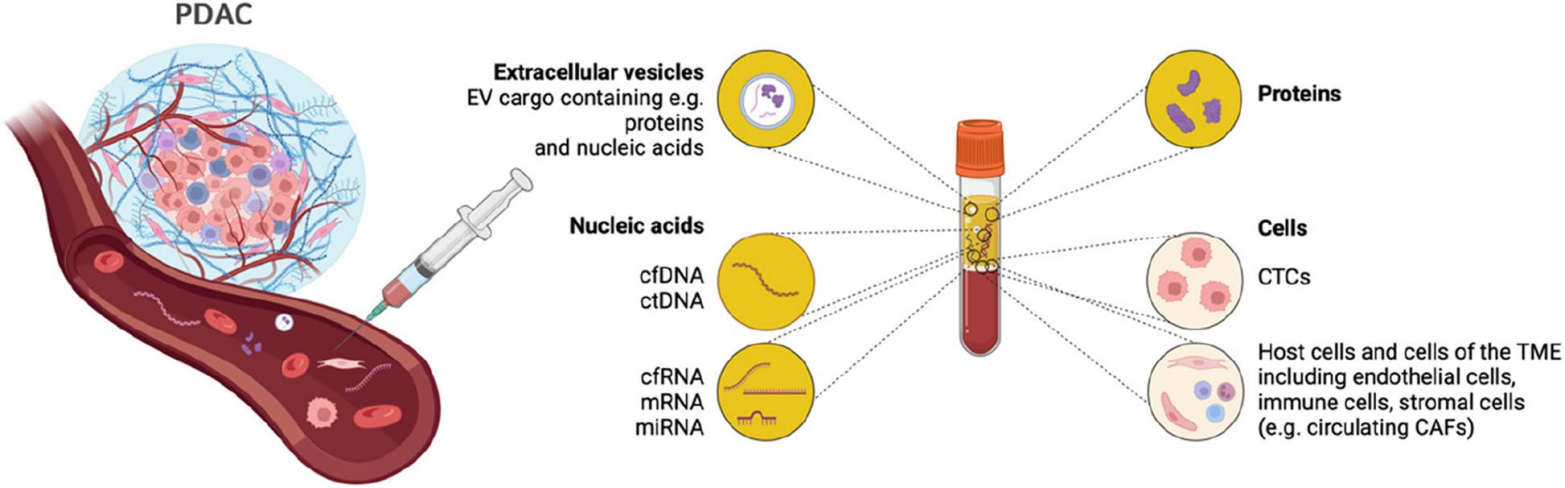
Overview of (blood-based) liquid biopsy analytes for the early detection of pancreatic cancer. The figure was created with BioRender.com under academic license

Liquid biopsy (LB) is a minimally invasive procedure that allows the sampling and analysis of body fluids, thus enabling cancer diagnosis, treatment monitoring, surveillance, and prognostication [9]. The samples can be obtained from various body fluids, including urine, saliva, cerebrospinal fluid, bone marrow, or blood [10]. In recent years, blood has been one of the most popular analytes, as blood sampling is easy, cost-effective, and repeatable [11]. Biomarkers that can be detected in the blood include circulating tumor cells (CTCs), circulating host cells, including cancer-associated fibroblasts (CAFs) or circulating endothelial cells (CECs), circulating cell-free RNA and DNA (cfRNA, cfDNA), extracellular vesicles (EVs) and proteins [12] **(**Fig. 1**)**. However, to date, no single biomarker or multimarker panel can reliably diagnose PDAC, especially in the early stages.

In this review, we summarize potential biomarkers and detection methods for blood-based liquid biopsy and discuss their implications for the early-stage detection of PDAC. Moreover, we review publications on multibiomarker panels for PDAC diagnosis and highlight their importance in the early diagnosis of pancreatic cancer.

## Methods

The literature search for this narrative review regarding multibiomarker panels was carried out in PubMed on 4th July 2024 via the following terms:

- ("biomarker panel*" OR "marker panel*" OR "multi biomarker*" OR "multi marker*" OR "marker combin*" OR "biomarker combin*") AND (pancrea*) AND (cancer OR carcinoma OR tumor* OR adenocarcinoma*) AND (sera OR serol* OR plasma OR blood OR "liquid biops*" OR "fluid biops*").

**Fig. 2:**
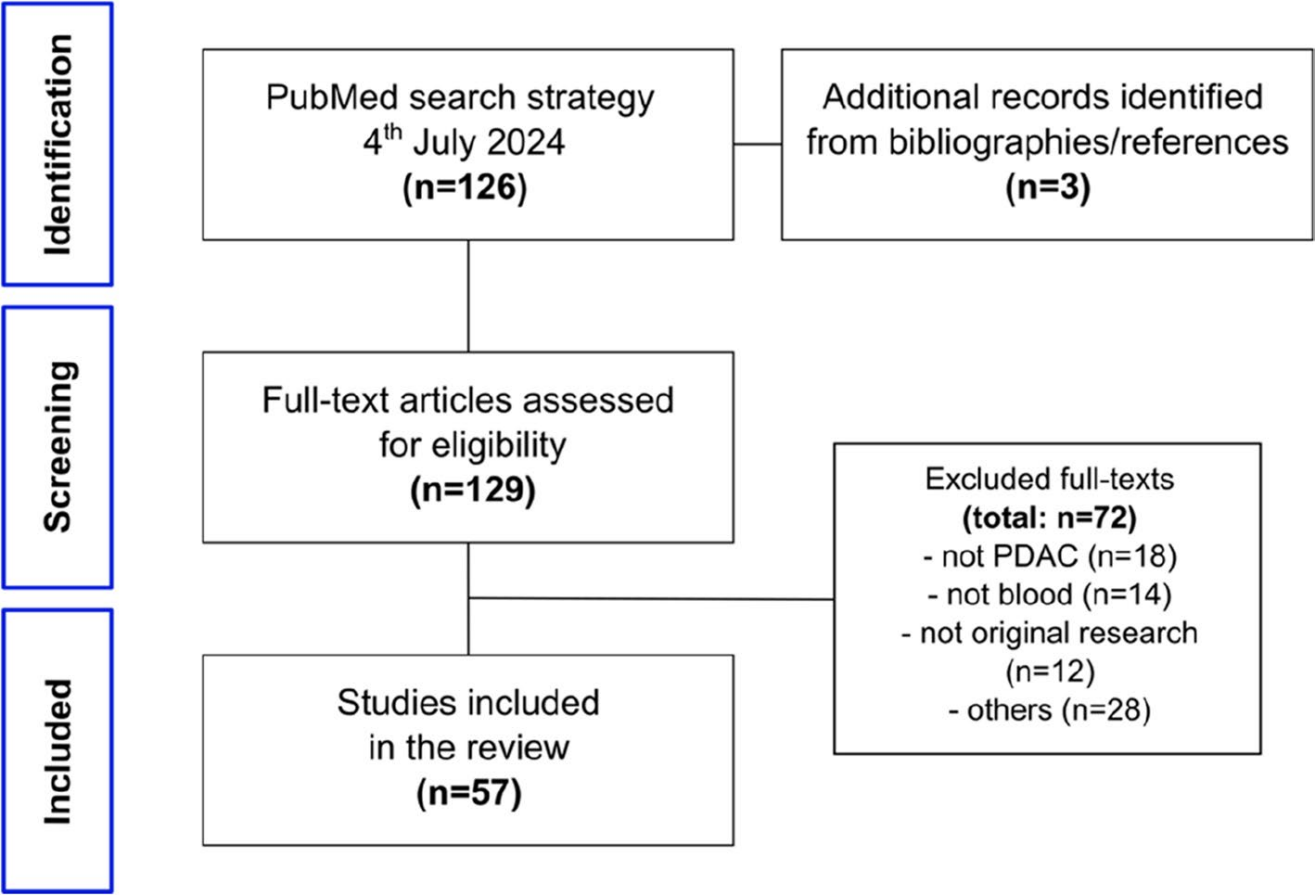
Overview of the results from the literature search and selection of included studies

The literature search was restricted to articles in English and yielded 126 papers. Reviews and case reports were excluded, as were studies that did not involve research on humans, PDAC, diagnosis, or blood-based liquid biopsy. According to these criteria, a total of 57 publications were further analyzed for this review. A flow chart can be found in Fig. 2. The data extracted from the publications included study details (author, year of publication, country), biomarker details (biomarkers used, detection method, fluid type), patient cohort details (patient numbers, stage, controls), and statistical details (sensitivity, specificity, area under the curve (AUC)). If separate data for early-stage PDAC (pathological AJCC/UICC stages I and II according to the 8th edition of the staging manual) and late-stage or all-stage PDAC diagnosis were provided, only the values for early-stage PDAC were included.

## Liquid biopsy biomarkers for PDAC

### Currently used tumor markers for PDAC

A commonly used serological biomarker for PDAC is carbohydrate antigen 19–9 (CA19-9), also referred to as sialyl Lewis-A, which currently represents the only FDA-approved marker. Increased levels of CA19-9 have been reported in PDAC patients compared with healthy individuals [13, 14]. The concentration of CA19-9 and its sensitivity as a diagnostic marker increase with increasing PDAC stage [15, 16], with the most pronounced increase detected between AJCC/UICC stage II and stage III. However, especially in early stages (e.g., stage I [17]), the level of CA19-9 is similar to that in various benign conditions, precancerous lesions, and other malignancies (e.g., colorectal cancer, gastric cancer, hepatocellular carcinoma), resulting in low specificity [14, 15]. With respect to its use for diagnostic purposes, it is important to consider that 6% of Caucasians and 22% of non-Caucasians who lack Lewis antigen A cannot produce CA19-9, subsequently leading to false-negative results [18]. Consequently, international guidelines do not recommend its use as a diagnostic method but rather as a longitudinal marker in patients with detectable CA19-9 at baseline [16]. Owing to the lack of robust biomarkers, various markers have been investigated as possible candidates with increased sensitivity and specificity for PDAC diagnosis. The following sections present an overview of cellular and acellular liquid biopsy-based biomarkers for PDAC diagnosis.

### Cellular biomarkers

One group of biomarkers analyzed in liquid biopsy is cells that have detached from their site of origin and entered the bloodstream. These can be derived from tumor or noncancerous host cells that are part of the tumor microenvironment (TME) (e.g., immune cells, fibroblasts, and endothelial cells) [19].

Tumor cells detected in the blood are referred to as circulating tumor cells (CTCs) [9]. CTCs are highly heterogeneous even if derived from the same patient [20]. Compared with classical diagnostic biopsies (e.g., fine needle biopsy), CTCs are able to provide a more representative image of tumor heterogeneity [21–23]. However, detecting CTCs is still challenging, as approximately one CTC is detectable among more than a million other blood cells (e.g., erythrocytes, leukocytes, and platelets), and CTCs have a short half-life of only 1–2.4 h [24, 25]. In addition to the number of CTCs, their genome, transcriptome, proteome and functional properties can be analyzed [26].

With respect to PDAC, a meta-analysis of 19 studies revealed that more than half of patients (707 out of 1320 patients analyzed) had detectable CTCs in their blood [27]. These patients had lower OS and PFS rates than CTC-negative patients did, highlighting the adverse prognostic effect of CTCs in PDAC patients. However, most patients in the studies included in the meta-analysis were in advanced tumor stages (stage III and IV: 61%), with only 31% in stage II and only 8% in stage I. The low number of CTCs, particularly in early PDAC stages, may lead to false negative results and low sensitivity [12, 28]. A potential explanation for the low CTC number in PDAC could be the filtration of CTCs in the liver before they reach the peripheral blood vessels and the reduced blood flow within the cancerous pancreas [29, 30]. This limits the analysis of CTCs as possible biomarkers for early diagnosis, but with the emergence of novel, more sensitive analysis techniques (e.g., in vivo CTC capture devices [31]) and techniques that allow processing of larger volumes [32, 33], this limitation may be overcome [34]. Despite the impaired sensitivity that could arise from the different methods used or the heterogeneity of CTCs, the specificity of CTCs for PDAC diagnosis has been reported in several studies to reach > 90% [35–37].

In PDAC, cells make up only a small part of the tumor, while the largest part is the dense stroma that forms the tumor microenvironment [38]. Compared with other solid tumors, PDAC has the most pronounced desmoplastic stroma reaction, which generates a physical barrier around the tumor, thereby impairing radical resection and increasing therapy resistance [39, 40]. Although the composition and structure of the stroma varies between patients, it consists of several main components [41]. Noncellular components, including glycoproteins, fibronectins, collagens, and enzymes, form the extracellular matrix (ECM). The cellular components include endothelial, immune, and stromal cells, including pericytes, and local cancer-associated fibroblasts (CAFs). These host cells can also detach from the TME, enter the bloodstream, and be analyzed as possible liquid biopsy biomarkers for PDAC (e.g., as circulating CAFs (cCAFs)).

CAFs are key components of the TME, and they are near or in direct contact with cancer cells [38]. The three different major types of CAFs, myofibroblast CAFs (myCAFs), inflammatory CAFs (iCAFs), and antigen-presenting CAFs (apCAFs), are associated with distinct functions and phenotypes [42]. These functions include the production of cytokines, chemokines, metabolites, enzymes, and ECM molecules to prevent or promote tumor growth [43]. cCAFs are found in the blood of patients with various tumors, including PDAC, where they are linked to a poorer prognosis in advanced stages [44–46]. Only one study examined cCAFs in six PDAC patients and reported an association between the presence of cCAFs and poorer clinical outcomes as well as lower OS rates at metastatic stages [47]. However, to our knowledge, there are currently no studies on the role of cCAFs in the early stages of PDAC, and such studies are crucial for identifying their suitability as biomarkers for early PDAC diagnosis.

### Circulating nucleic acids

Circulating tumor DNA (ctDNA) is a type of cell-free DNA (cfDNA) derived from tumor cells that can be found in the bloodstream. ctDNA can be released by cells undergoing apoptosis and necrosis or can be actively transported through the cell membrane [48]. Since only up to 1% of the cfDNA in the blood of early-stage patients originates from tumors, most detectable circulating nucleic acids are cfDNA from noncancer cells, which limits the ability to detect ctDNA [49, 50]. The amount of ctDNA in the blood varies between different tumors and increases up to 40% in advanced tumor stages [50, 51]. The half-life of ctDNA is estimated to be between 16 and 114 min, which makes isolation more challenging [52, 53]. ctDNA can be detected due to specific alterations in the tumor and can be examined for mutations, DNA integrity, gene fusion, copy number variation, or methylation status [54, 55]. The high concordance between mutations in ctDNA and those in tumor tissue makes it suitable as a biomarker that provides information about the primary tumor even if it is inaccessible [56].

Analysis of genomic aberrations in all-stages PDAC tissue revealed a panel of four genes, namely, *KRAS*, *CDKN2A*, *TP53,* and *SMAD4,* with mutation frequencies of 90%, 90%, 70%, and 55%, respectively, as the main genomic drivers of PDAC [57–61]. Interestingly, mutations in these genes can be detected in preneoplastic PanIN lesions; notably, *KRAS* is the first event, and subsequent alterations in *CDKN2A*, *TP53,* and *SMAD4* can be detected in higher-grade PanINs [62, 63]. These mutations lead to increased proliferation, dysregulation of the cell cycle and an impaired DNA damage response [59]. As *KRAS* mutations are among the initiating mutations during the development of PDAC, *KRAS* mutations are interesting biomarkers for the early diagnosis of PDAC [64]. On the basis of the molecular profile of PDAC, several studies have used these mutations for ctDNA detection [64]. However, germline mutations in cfDNA or clonal hematopoiesis of indeterminate potential (CHIP) in noncancerous cells, especially related to *KRAS* (approximately 30%) [65], may lead to false-positive results and should be considered in related evaluations [55, 66, 67].

A meta-analysis of seven retrospective studies on the utility of ctDNA as a liquid biopsy biomarker revealed a sensitivity of 64% (95% CI 0.58–0.70), a specificity of 92% (95% CI 0.88–0.95), and an AUC of 0.9478 across all PDAC stages [28]. With approximately only one molecule of ctDNA in every 5 mL of plasma, the moderate sensitivity is presumably the result of minute amounts of released ctDNA, especially in the early tumor stages, when the rates of apoptosis and necrosis are lower [68, 69].

In addition to somatic cancer alterations, epigenetic traits (e.g., methylation, fragmentation) can also be examined in ctDNA. Epigenetic alterations have recently received much attention, as they may also provide tissue-specific information that helps to determine the organ in which the tumor originates. Nicholson et al*.* analyzed the cfDNA methylation pattern in a prospective study of 5,461 participants with suspected cancer and were able to detect different tumors with a sensitivity of 66.3% in all stages and 24.4% in stage I patients, with a specificity of 98.4% [70]. A recent publication by García-Ortiz et al*.* reviewed studies analyzing the ctDNA methylation status in PDAC and concluded that the use of a single epigenetic biomarker does not allow for the diagnosis of early-stage PDAC and suggested that a multimarker panel would be more efficient [71]. Moreover, fragmentomic approaches focusing on fragment size, fragment ends, and end motifs can reveal differences between ctDNA and cfDNA [72]. ctDNA from cancer patients is shorter than nontumor cfDNA, and its feautures differ between different tumor entities, which enables the identification of the tissue of origin [73, 74]. Cristiano et al. were able to detect pancreatic tumors with a sensitivity of 71% at a specificity of 95% on the basis of the cfDNA fragment size [54]. These studies underscore the promising value of cfDNA-based approaches that are independent of the presence of genomic signatures.

In addition to cfDNA, cancer cells also release cell-free RNA (cfRNA) into the circulation [75, 76]. In addition to intracellular coding messenger RNAs (mRNAs), which are required for protein synthesis, noncoding RNAs, including microRNAs (miRNAs), are potential biomarker candidates [77]. cfRNAs are highly stable, as they are typically packed in extracellular vesicles or attached to lipid or protein complexes rather than circulating freely in the bloodstream [78–82].

miRNAs are noncoding, single-strand RNAs with an average length of 22 nucleotides that are highly evolutionarily conserved among various species [83]. miRNAs can regulate their target mRNAs at the posttranscriptional level by affecting their translation and stability [84–86]. In cancer patients, altered expression of miRNAs has been reported [87]. Numerous studies examining the role of various miRNAs as LB biomarkers in PDAC have been conducted and reviewed elsewhere [57]. In a meta-analysis, Peng et al. examined miRNAs from 46 studies involving 4,326 patients with pancreatic cancer [88]. The diagnostic performance of miRNA panels, which included 4.5 miRNAs on average (range: 2–12 miRNAs), was compared to that of single miRNAs and interestingly exhibited no significant diagnostic benefit. The combined results yielded a sensitivity of 79% (0.77–0.81), a specificity of 77% (0.75–0.79), and an AUC of 0.85 (0.81–0.87). Considering only early-stage PDAC (up to stage IIA), the diagnostic value decreased slightly to a sensitivity of 79% (0.76–0.82), a specificity of 74% (0.68–0.79), and an AUC of 0.81 (0.77–0.84) [88].

### Proteins

Proteins are important for communication between cancer cells and host cells in the TME [89]. Proteins can be located on the membrane surface of cells but can also be secreted in vesicles or released into the circulation [90]. A wide range of different circulating proteins, including cytokines, chemokines, carbohydrate antigens, growth factors, inflammatory factors, glycoproteins, and apolipoproteins, orchestrate numerous biological processes [91–93]. Proteins released by cancer cells can regulate the development and progression of cancer by promoting invasion and metastasis [89]. Several proteins are up- or downregulated in the blood of PDAC patients compared to that of healthy donors or benign tumor patients [94]. Hence, many circulating proteins have been analyzed as potential biomarkers for PDAC and are reviewed in more detail elsewhere [95]. However, interestingly, the sensitivity and specificity of most single proteins do not exceed those of CA19-9 [5]. A meta-analysis by Kane et al*.* compared 250 prospective and retrospective studies published before July 2020 on all stages of PDAC; the results revealed an AUC of 0.85 for CA19-9 alone and 0.783 for novel single biomarkers [96].

### Extracellular vesicles

Extracellular vesicles (EVs) are lipid-bound and secreted particles that comprise three classes of vesicles: exosomes (30–150 nm), microvesicles (50–1000 nm), and apoptotic bodies (500–5000 nm). EVs can be released by several cell types, including neurons, epithelial cells, and fibroblasts, as well as cancer cells [97–99]. Exosomes are particularly interesting for liquid biopsy approaches because they contain many molecules, including lipids, metabolites, nucleic acids (e.g., miRNAs and mRNAs), and proteins, that are protected from degradation by the EV membrane [100]. As exosomes transfer these molecular cargoes to recipient cells through cell‒cell interactions or even over large distances, e.g., between different organs, they are important for cellular communication [101].

Exosomes influence tumor malignancy by regulating the tumor microenvironment, angiogenesis, tumor growth, invasion, and metastasis, including epithelial‒mesenchymal transition, immunomodulation, and chemoresistance [98, 102–106]. Moreover, cancer cells, including PDAC cells, secrete more exosomes than noncancerous cells [107, 108].

In a meta-analysis on the potential utility of extracellular vesicle cargo as biomarkers for PDAC, Jia et al. examined 39 studies including 2,037 PC patients [109]. Seventeen studies on EV RNAs, 16 on EV proteins, and 16 on EV biomarker panels were evaluated across all tumor stages. The most reported molecules were the EV RNAs miR-21 and miR-10b and the EV proteins GPC1 and EphQ2. A sensitivity of 84% (95% CI: 81–86%) and a specificity of 89% (95% CI: 86–91%) were obtained from the pooled values of EV RNAs and EV proteins. In contrast to analysis of the previously described biomarker types, the analysis of EVs in early PDAC stages I and II led to an increased sensitivity of 90% (95% CI: 87–93%) and specificity of 94% (95% CI: 92–95%). Interestingly, EVs as markers seem to perform at least as well and possibly even better in earlier stages (although only modestly with almost overlapping CIs) than in advanced stages [109]. One potential explanation could be that patients with advanced PDAC suffer from dysregulated EV secretion due to cancer-related effects, including cachexia and dysregulated metabolic processes. These findings indicate that exosomes and their cargo are potential biomarkers for the early diagnosis of PDAC.

## Multimarker analysis

Numerous single biomarkers for the diagnosis of PDAC have been investigated, as a single marker can facilitate diagnostic assay development and implementation in routine clinical practice. However, the investigated markers have low sensitivity and specificity for diagnosis. Considering the high degree of patient diversity and tumor heterogeneity, a multimarker panel can provide complementary value and seems to perform better than single biomarkers do.

The literature search yielded 57 papers that analyzed multibiomarker panels in blood to diagnose PDAC. As some publications included two different panels, the total number of multibiomarker panels assessed was 63. Among these panels, 57 included proteins, 10 included RNA, 6 included EVs, 4 included cfDNA, 2 included metabolites, and 1 included CTCs. An overview of these studies [16, 110–165] can be found in Table 1.

**Table 1:** Overview of multimarker studies for the (early) diagnosis of PDAC using liquid biopsy

Biomarker	SN [%]	SP [%]	AUC	Total patients [N] (female)	Stage	Control	Detection method	Fluid	Country	Reference
**Protein panels—all PC stages**										
CA19-9 + OPN + CHI3L1	93	81	N/A	52 PDAC (N/A)	II, III	43 HC	PLA	plasma	USA	Chang, 2009 [110]
CA19-9 + TFPI	84	90	0.94	37 PDAC (15)	I:0, II:1, III:2, IV:9, N/A:25	15 HC	ELISA	plasma	USA	Balasenthil, 2011 [111]
CA19-9 + ICAM-1 + OPG	78	94	0.91	173 PDAC (95)	I:4, II:37, III:20, IV:41, N/A:71	120 HC	MIA	serum	USA	Brand, 2011 [112]
CA19-9 + CEA + TIMP-1	71	89	0.83	173 PDAC (95)	I:4, II:37, III:20, IV:41, N/A:71	70 BD	MIA	serum	USA	Brand, 2011 [112]
CA19-9 + Cathepsin D + MMP-7	89	77	0.91	139 PDAC (50)	N/A	74 HC, 72 CP	MIA	serum	South Korea	Park, 2012 [113]
CA19-9 + AACT + THBS1 + HPT	77	90	0.92	37 PC (21)	IA:3, IB:1, IIA:6, IIB:8, III:2, IV:17	30 HC, 30 DM, 30 PC, 30 CP, 22 OJ	ELISA	serum	USA	Nie, 2014 [114]
CA19-9 + CEA + Cyfra 21–1	30	95	0.68	135 PC (56)	N/A	540 HC	MIA	serum	USA	Nolen, 2014 [115]
CA19-9 + IL-6 + IP-10 + PDGF	81	92	0.88	43 PDAC (19)	resectable:31, advanced:12	7 BD	ELISA + cytokine assay	serum	UK	Shaw, 2014 [116]
CA19-9 + IL-6 + IP-10 + IL-8	N/A	N/A	0.91	43 PDAC (19)	resectable:31, advanced:12	17 CP	ELISA + cytokine assay	serum	UK	Shaw, 2014 [116]
CA19-9 + AACT + THBS1 + SAAV peptide	100	96	0.99	26 PDAC (15)	IA:2^‡^, IB:1^‡^, IIA:3^‡^, IIB:7^‡^, IV:24^‡^	27 HC	ELISA + MS	serum	USA	Nie, 2014 [117]
CA19-9 + sLeX	76	78	N/A	109 PC (N/A)	N/A	30 BD	ELISA	plasma	USA	Tang, 2015 [118]
CA19-9 + APOE + ITIH3 + APOA1 + APOL1	95	94	0.99	40 PC (N/A)	N/A	34 HC	ELISA + MS	serum	China	Liu, 2017 [119]
CA19-9 + FVIII + fibrinogen + albumin + conjugated bilirubin + ALP	N/A	N/A	0.95	67 PDAC (36)	I-III	18 IPMN	coagulation assay	plasma	Finland	Mattila, 2018 [120]
CA19-9 + TSP-2	91	99	0.95	263 PDAC (140)	I-II:57, III-IV:206	230 IAR	ELISA	serum	Taiwan	Peng, 2019 [121]
CA19-9 + sTRA	54	95	0.86	71 PDAC (31)	I:17, II:40, III:5, IV:9	20 HC, 9 PC, 15 CP, 8 BD, 24 DM	ELISA	plasma	USA	Staal, 2019 [122]
CA19-9 + AGP1	89	97	0.96	52 PC (23)	N/A	34 HC	immunoturbidimetry, ELISA	serum	Sweden	Zhou, 2019 [123]
CA19-9 + CA125 + CEA + APOA1 + APOA2 + TTR	93	96	0.99	60 PDAC (18)	I:18, II:24, III:10, IV:8	191 HC	immunoturbidimetry, ELISA	plasma	South Korea	Kim, 2020 [124]
CA19-9 + CLU + C5 + KLKB1 + PPBP + IFRD1 + IGFBP2 + ICAM1 + C4BPA + PTPRJ + ECM1 + VIM + C4BPB + SERPINA5 + TTR	86	97	0.97	65 PDAC (27)	IA:1, IB:0, IIA:11, IIB:15, III:10, IV:93	59 HC, 13 BD	MS	plasma	South Korea	Kim, 2021 [125]
CA19-9 + S100A2 + S100A4 + CA125	N/A	N/A	0.91	120 PDAC (59)	I + II:9, III + IV:111	80 HC	ELISA	serum	Australia	Mehta, 2021 [126]
CA19-9 + VWF + MUC16 + THBS2 + FASLG + CEACAM5 + TLR3 + HGF + HRT + TNFRSF19 + CTSV + VEGEFA + FCRLB + ERBB4 + ANXA1 + ERBB2 + OCP + NT5E + CCN1 + TGFA	92	90	0.91	218 PDAC (N/A)	N/A	249 NC	ELISA, OLINK	serum	UK	Nené, 2023 [127]
CA19-9 + CEA + CA125	82	85	0.90	65 PDAC (N/A)	N/A	700 HC	ECLIA	serum	China	Cao, 2023 [128]
IL-7R + PLD4 + ID3	73	79	0.76	33 PDAC (N/A)	N/A	80 BD	RT-PCR	plasma	USA	Jang, 2023 [129]
**Protein panels—early PC stages (stage I-II)**										
CA19-9 + SYCN + REG1B	39	95	0.87	20 PDAC (14)	I, II	92 HC	ELISA	plasma	Canada	Makawita, 2013 [130]
CA19-9 + SYCN + REG1B	68	95	0.92	40 PDAC (18)	I, II	47 HC	ELISA	serum	Canada	Makawita, 2013 [130]
CA19-9 + CA125 + LAMC2	78	63	0.76	27 PDAC (N/A)	IA:5, IB:5, IIA:17	17 BD	ELISA	serum	Canada	Chan, 2014 [16]
CA19-9 + sLeX + type 1 N-acetyl-lactosamine	80	84	N/A	50 PDAC (27)	I:3, II:47	10 BD	ELISA	plasma	USA	Tang, 2016 [131]
CA19-9 + IGFBP2 + IGFBP3	N/A	N/A	0.90	38 PDAC (14)	I:4, II:34	65 HC	MS	plasma	Japan	Yoneyama, 2016 [132]
CA19-9 + LRG1 + TIMP1	67	95	0.89	39 PDAC (18)	IA:6, IB:10, resectable (no TNM data):23	82 HC	ELISA, MS	plasma	USA	Capello, 2017 [133]
CA19-9 + TFPI + TNC-FN III-C	75	82	0.83	98 PDAC (55)	IA:7, IB:8, II:1, IIA:40, IIB:42	61 HC	ELISA	plasma	USA	Balasenthil, 2017 [134]
CA19-9 + MUC5AC	75	83	0.84	63 PC (N/A)	IA:2, IB:4, IIA:18, IIB:46	35 HC, 30 BD, 43 CP	ELISA, RIA	serum	USA	Kaur, 2017 [135]
CA19-9 + THBS2	N/A	N/A	0.96	88 PDAC (43)	I:4, IA:2, IB:4, II:37, IIA:15, IIB:26	140 HC	ELISA	plasma	USA	Kim, 2017 [136]
CA19-9 + LRG1 + TTR	78	94	0.91	50 PDAC (N/A)	I:4, II:46	68 HC, 21 BD	ELISA	plasma	South Korea	Park, 2017 [137]
CA19-9 + MMP7	N/A	N/A	0.98	25 PDAC (N/A)	IA:2, IB:1, IIA:22	131 HC	ELISA, MIA	plasma	Italy	Resovi, 2018 [138]
CA19-9 + CCN2 + Col4 + FN + PLG	N/A	N/A	0.92	25 PDAC (N/A)	IA:2, IB:1, IIA:22	30 CP	ELISA, MIA	plasma	Italy	Resovi, 2018 [138]
CA19-9 + POSTN + CA242	92	97	0.98	38 PDAC (N/A)	IA:3, IB:5, IIA:30	37 HC	ELISA	serum	China	Dong, 2018 [139]
CA19-9 + MIA	N/A	N/A	0.86	96 PDAC (N/A)	IA:13, IB:18, IIA:17, IIB:48	68 CP	MIA	serum	USA	Song, 2019 [140]
CA19-9 + MIC-1	N/A	N/A	0.81	96 PDAC (N/A)	IA:13, IB:18, IIA:17, IIB:48	63 IPMN	MIA	serum	USA	Song, 2019 [140]
CA19-9 + TFF1 + TFF2 + TFF3	33	100	0.85	18 PC (N/A)	I, II	8 BD	ELISA	serum	USA	Jahan, 2019 [141]
CA19-9 + MUC5AC	77	89	0.89	30 PDAC (14)	N/A	34 HC, 29 BD, 35 Choledocholithiasis, 25 CP	ELISA, EIA	serum	China	Zhang, 2020 [142]
CA19-9 + APOA4 + CD14 + CLEC3B + GSN + HRG + ITIH3 + KLKB1 + LRG1 + SERPINF1 + SERPING1 + TIMP1	80	80	0.85	50 PDAC (N/A)	IA:3, IB:1, IIA:11, IIB:35	49 DM	ELISA, MS	plasma	USA	Peng, 2020 [143]
CA19-9 + LRG1 + TTR	92	88	N/A	248 PDAC (N/A)	I:20, II:228	347 HC/ gallstones/ cholecystitis	ELISA	plasma	South Korea	Choi, 2021 [144]
CA19-9 + LRG1 + TTR	94	91	N/A	657 PDAC (N/A)	I:39, II:618	609 HC	ELISA	plasma	South Korea	Lee, 2023 [145]
CA19-9 + CA125	72	80	0.81	56 PDAC (N/A)	IA:5, IB:6, IIA:9, IIB:36	53 IPMN	ECLIA	serum	USA	Song, 2021 [146]
EV proteins: CA19-9 + CA125 + Cathepsin D + Ferritin + sE-selectin + IGFBP3 + MIA + CA 15–3 + sFAS + TIMP1 + sNeuropilin-1 + MPO + bHCG	96	100	N/A	47 PDAC (33)	I:22, II:25	184 HC	IA	plasma	USA	Hinestrosa, 2022 [147]
CA19-9 + CEA + ALCM + ANG + AXL + BAG3 + BSG + CEACAM + COL18A1 + EPCAM + HA + HP + ICAM + IGFBP2 + IGFBP4 + LCN2 + LRG1 + MMP2 + MMP7 + MMP9 + MSLN + PARK7 + PPBP + PRG4 + SPRCL1 + SPP1 + TGFB1 + THBS1 + TIMP1 + TNFRSF1A + WEGFC	63	97	0.94	30 PDAC (12)	I:4: IIA:8, IIB:18	103 HC	ELISA	serum	USA	Firpo, 2023 [148]
CA19-9 + PIGR + vWF	N/A	N/A	0.98	28 PDAC (N/A)	IA:4, IB:5, II:2, IIB: 17	28 HC	ELISA	serum	Australia	Byeon, 2024 [149]
**DNA panels**										
* BNC1* + *ADAMTS1* methylation	81	85	N/A	10 PC (N/A)	I:10	26 HC	PCR, MOB	serum	USA	Yi, 2013 [150]
* BNC1* + *ADAMTS1* methylation	95	92	0.95	37 PDAC (13)	I:8, IIA:9, IIB:20	95 HC, 8 pancreatitis	PCR	plasma	USA	Eissa, 2019 [151]
**RNA panels**										
miR-642b + miR-885-5p + miR-22	91	91	0.97	11 PDAC (6)	II	11 HC, 11 IAR	qRT-PCR	plasma	USA	Ganepola, 2014 [152]
EV miRNA: miR-93-5p + miR-339-3p + miR-425-5p + miR-425-3p	80	95	0.89	15 PDAC (2)	I:3, II:2, III:3, IV:7	19 C	qRT-PCR	plasma	USA	Makler, 2023 [153]
miR-28-3p, miR-143-3p, and miR-151a-3p	N/A	N/A	0.81	14 PDAC (N/A)	I:8, II:6	107 C	qRT-PCR	plasma	China	Yang, 2024 [154]
**multi-omics panels—all PC stages**										
CD44v6 + Tspan8 + EpCAM + MET + CD104 + miR-1246 + miR-4644 + miR-3976 + miR-4306	100	80	N/A	131 PC (76)	N/A	30 HC, 20 BD, 25 C, 12 non-PC	qRT-PCR, flow cytometry	serum	Germany	Madhavan, 2015 [155]
CA19-9 + MIC-1 + miR-21	88	99	0.97	82 PC (35)	I:1, II:7, III:51, IV:23	88 HC	RT-PCR, ELISA	plasma	China	Yuan, 2016 [156]
CA19-9 + MIC-1 + miR-25	84	100	0.97	82 PC (35)	I:1, II:7, III:51, IV:23	88 HC	RT-PCR, ELISA	plasma	China	Yuan, 2016 [156]
CA19-9 + THBS2 + cfDNA quantification	87	92	0.94	52 PDAC (26)	I:14, II:17, III:21	15 IPMN, 32 pancreatitis	ELISA, Qubit	plasma	Germany	Berger, 2019 [157]
CA19-9 + EV: miR200b + miR200c	92	100	0.97	56 PDAC (20)	IIA:4, IIB:14, III:22, IV:24	22 HC + 11 CP	PCR, western blot	serum	Germany	Reese, 2020 [158]
CA19-9 + CTC quantification	91	91	0.95	80 PDAC (29)	I + II:40, III + IV:40	34 HC, 32 AP, 22 BD	NE-imFISH, ECLIA	whole blood, plasma	China	Chen, 2022 [159]
**Multi-omics panel—early PC stages (stage I-II)**										
CA19-9 + CEA + HGF + OPN + ctDNA: *KRAS* mutation	64	100	N/A	221 PDAC (100)	I:29, II:192	182 HC	PCR, MIA	plasma	multi-center	Cohen, 2017 [160]
CA19-9 + LRG1 + TIMP1 + acetylspermidine + diacetylspermine + indole-derivative + two lysophosphatidylcholines	90	88	0.92	39 PDAC (18)	IA:6, IB:10, resectable (no staging data):23	82 HC	ELISA, MS	plasma	USA	Fahrmann, 2019 [161]
CA19-9 + cf miRNA: miR30c-5p, miR340-5p, miR335-5p, miR23b-3p, miR142-3p + EV miRNA: miR145-5p, miR200b-3p, miR429, miR1260b, miR145-3p, miR216b-5p, miR200a-3p, miR217-5p	84	99	0.99	91 PDAC (N/A)	I:36, II:55	67 HC	qRT-PCR, ELISA	plasma + serum	USA, Japan, South Korea	Nakamura, 2022 [162]
CA19-9 + proline + creatine + palmitic acid	86	82	0.95	22 PDAC (11)	IA:2, IB:6, IIA:2, IIB:12	27 HC	MS	serum	China	Zhao, 2023 [163]
CA19-9 + hsa_circ_0060733 + hsa_circ_0006117 + hsa_circ_0064288 + hsa_circ_0007895 + hsa_circ_0007367	77	95	0.94	63 PDAC (N/A)	I, II	46 HC	qRT-PCR	plasma	USA	Xu, 2023 [164]
EV: CD63 + GPC-1 + HER2 + snRNA U6 + GPC-1 mRNA + miR-10b	N/A	N/A	0.93	15 PDAC (8)	IA:1, IB:9, IIA:3, IIB: 2	15 HC	Co-PAR	plasma	China	He, 2024 [165]

*Abbreviations*: *AC* Acute pancreatitis, *AUC* Area under the curve, *BD* Benign disease, *C* Control (undefined), *Co-PAR* Codetection platform of Proteins and RNAs, *CP* Chronic pancreatitis, *ECLIA* electrochemiluminescence-based immunoassay, *EIA* Enzyme immunoassay, *ELISA* Enzyme-linked immunosorbent assay, *DM* Diabetes mellitus, *HC* Healthy control, *IA* Immunoassay, *IAR* Individual at risk, *IPMN* Intraductal papillary mucinous neoplasm, *MIA* Magnetic immunoassay, *MS* Mass spectrometry, *N/A* Not available, *PC* Pancreatic cancer, *PCR* Polymerase chain reaction, *PDAC* Pancreatic adenocarcinoma, *SN* Sensitivity, *SP* Specificity. ^‡^ As only TNM stage data were present in the original study, staging was assessed according to the 8th edition of the UICC staging manual

Many publications started by analyzing single biomarkers and later combined them with one or more other biomarkers. The addition of biomarkers led to increased sensitivity and specificity and improved AUC values in these studies. For example, Capello et al*.* calculated an AUC of 0.730 for TIMP1, 0.832 for LRG2, and 0.821 for CA19-9 to distinguish early-stage PDAC patients from healthy controls [133]. The combination of all three protein markers increased the AUC to 0.887, and adding 5 metabolites to the protein panel further increased the AUC to 0.924 [161]. This observation was quantified in the above-mentioned meta-analysis by Kane et al*.* [96]. The pooled AUC for studies with single biomarkers was 0.803, which was significantly lower than the multibiomarker panel AUC of 0.898. However, this analysis was performed on all stages of PDAC and did not focus particularly on the early stages.

The investigated biomarkers were combined with CA19-9 analysis in 55 of the 63 studies, and only 8 studies did not include CA19-9 in their panel [129, 150–155, 165]. Adding CA19-9 to other biomarkers improved the diagnostic power. For example, Dong et al*.* examined the proteins POSTN and CA242 in early-stage PDAC patients versus healthy controls and reported an AUC of 0.92 for their combination [139]. The addition of CA19-9 to the panel increased the AUC to 0.98.

**Fig. 3:**
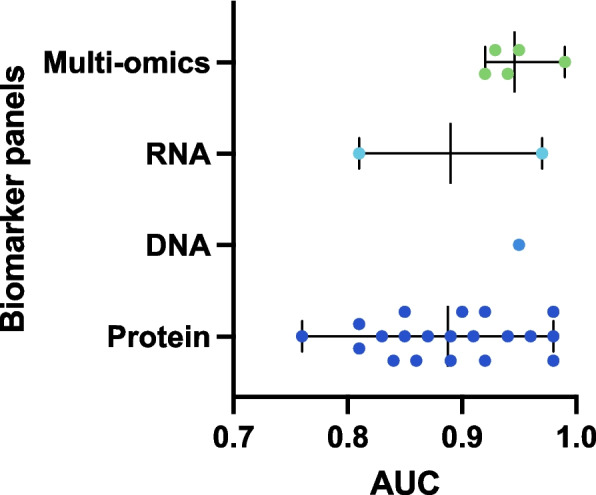
Distribution of the AUC values of multibiomarker panels in identifying stage I and II PDAC patients. The AUC values derived from Table 1 for the multibiomarker panels for early-stage PDAC are plotted. The figure includes multimarker panels consisting of combinations of proteins (20 studies), DNA (1 study), RNA (2 studies) or multiomic markers (5 studies)

Most biomarker panels included only protein markers (46 out of 63 studies) that were analyzed directly from the blood or isolated from EVs. In the studies on early-stage PDAC, the AUCs ranged from 0.76–0.98 (Fig. 3). The protein panels consisted of two proteins in 7 studies, three proteins in 11 studies, and four or more proteins in 5 studies, although the number of proteins did not appear to directly correlate with the reported AUC. The biomarker panels included numerous different proteins, whereas only some proteins, including CA19-9, CEA, and MUC5AC, were found in several panels. Hinestrosa et al*.* isolated EVs from the blood of early PDAC patients and healthy controls and analyzed a panel of 13 proteins within EVs, resulting in a sensitivity of 95.7% and specificity of 99.5% [147].

Only five studies focused on other types of biomarkers, namely, cfDNA [150, 151] or RNA [152–154]. Eissa et al*.* analyzed the cfDNA methylation pattern of the *BNC1* and *ADAMTS1* genes and reported the ability to distinguish early-stage PDAC patients from mixed controls, with an AUC of 0.95 [150, 151]. Ganepola et al*.* compared the miRNAs miR-642b, miR-885-5p, and miR-22 between stage II PDAC patients and healthy controls as well as high-risk patients, resulting in an AUC of 0.97 [152]. A prospective study analyzing the 2’-O-methylated miRNAs miR-28-3p, miR-143-3p, and miR-151a-3p in 135 individuals was performed by Yang et al*.* The panel identified 20 out of 28 early-stage PDAC patients, resulting in an AUC of 0.81 [154].

Furthermore, several studies have investigated multiomic panels by combining analyses of CTCs, cfDNA, metabolites, or miRNAs with proteins [155–165]. The only reviewed study that included CTCs for PDAC diagnosis was performed by Chen et al*.* [159]. The authors isolated and quantified CTCs from whole blood and added CA19-9 analysis to distinguish all-stage PDAC patients from mixed controls with an AUC of 0.95. Two studies analyzed cfDNA in combination with different proteins: Cohen et al*.* focused on four proteins (CA19-9, CEA, HGF, OPN) and *KRAS* mutations in cfDNA, distinguishing early-stage PDAC patients from healthy controls with a sensitivity of 64% and a specificity of 99.5% [160]. In a different cfDNA analysis approach, Berger et al*.* quantified ctDNA and analyzed CA19-9 and THBS2 to differentiate between all-stage PDAC, IPMN and pancreatitis, with an AUC of 0.94 [157]. Metabolites were included in the studies of Fahrmann et al*.* and Zhao et al*.* to distinguish early-stage PDAC patients from healthy controls. The multibiomarker panel of Fahrmann et al*.* consisted of five metabolites (acetylspermidine, diacetylspermine, an indole-derivative, and two lysophosphatidylcholines) and three proteins (CA19-9, LRG1, and TIMP1), resulting in an AUC of 0.924 [161]. Zhao et al*.* combined three metabolites (proline, creatine, and palmitic acid) with CA19-9, and this panel had an AUC of 0.949 [163]. Seven studies involved combined analysis of proteins with cell-free RNAs or miRNAs isolated from EVs [155, 156, 158, 162, 164, 165]. Nakamura et al*.* combined CA19-9 with 5 cell-free miRNAs (miR30c-5p, miR340-5p, miR335-5p, miR23b-3p, and miR142-3p) and 8 EV-derived miRNAs (miR145-5p, miR200b-3p, miR429, miR1260b, miR145-3p, miR216b-5p, miR200a-3p, and miR217-5p) [162]. This biomarker panel had an AUC of 0.99 for distinguishing early-stage PDAC patients from healthy controls, indicating that it was the most precise diagnostic panel among all reviewed studies on early-stage PDAC. However, there were some limitations to this study, such as the modest sample size (*n* = 91) and the lack of age-matched control groups, which need to be addressed before the biomarker panel can be applied in the clinic.

The analyzed biomarkers were tested on early-stage PDAC patient samples in 34 of the 63 studies and had AUCs in the range of 0.76–0.99 (Fig. 3). The protein panels had the lowest mean AUC of 0.89, and the range of AUC values was the widest. The multiomic panels had the highest mean AUC of 0.95, with a small range from 0.92–0.99, indicating that combining different omic biomarkers yields greater statistical power. However, compared with single markers or panels with only one type of marker (e.g., protein), multimarker panels, particularly multiomic panels, involve more elaborate integrative assays with potentially increased development time and greater complexity.

These studies indicate that several biomarkers perform well in detecting early-stage PDAC. However, for these markers to be used for the screening of risk groups, the sensitivity and specificity need to be increased to minimize the number of false positive and negative diagnoses. In particular, high sensitivity is difficult to reach, as PDAC shares numerous biomarkers and mutations (e.g., RAS mutations) with other diseases (e.g., colorectal cancer), benign diseases of the pancreas (e.g., pancreatitis) or its precancerous lesions (e.g., IPMN), and these other diseases have higher prevalence in the general population than PDAC [166, 167]. Diseased controls (e.g., those with precancerous conditions, pancreatitis, and pancreatic cysts) and individuals at risk for developing PDAC should be included in studies to minimize false-positive rates and gain further knowledge of the molecular tumorigenesis of PDAC. On the basis of these assumptions, screening for PDAC in the general population could be implemented in pancancer screening efforts rather than as a specific test for PDAC. A panel of multiple markers could be used to screen for several cancer types at the same time and therefore be used on a broader group of individuals. Two multimarker panel-base tests, CancerSEEK [168] and Galleri (GRAIL) [169–172], were developed to detect the early stages of multiple tumors, including PDAC. The CancerSEEK multimarker panel includes ctDNA and eight proteins (CA-125, CEA, CA19-9, PRL, HGF, OPN, MPO, and TIMP-1) to detect ovarian, liver, stomach, pancreatic, esophageal, colorectal, lung and breast tumors. Currently, the CancerSEEK test is only used in clinical trials (NCT04213326). The Galleri test by GRAIL, which is already commercially available, analyzes the whole-genome methylation of cfDNA to detect signals of more than 50 cancer types. In an independent validation set of 4,077 individuals, the test was able to identify 35 of 41 patients with early-stage pancreatic cancer, resulting in a sensitivity of approximately 60% at 99.5% specificity [171]. Moreover, in another prospective study of 6,662 participants, the Galleri test detected a suspicious positive cancer signal in 92 cases [172]. After 12 months of follow-up, 35 of these 92 participants (38%) were confirmed to have a true positive cancer diagnosis, and 6,235 of the 6,549 (95.5%) participants without a cancer signal had true negative results, highlighting the feasibility of multicancer early detection (MCED) testing. Moreover, for the first time, this study assessed the subsequent diagnostic pathways and time to diagnostic resolution [172]. Notably, these studies by Schrag et al., are highly important for emphasizing the clinical utility of screening approaches (e.g., MCED tests), even in nonrisk groups.

Another study on the methylation of cfDNA, the THUNDER study, yielded high sensitivity for advanced stages but only approximately 35% sensitivity for stages I and II, with 98.9% specificity for detecting pancreatic tumors [173]. Although the specificity of the tests is sufficient, the sensitivity for early-stage diagnosis is lower than that of the PDAC-specific panels outlined in this review and therefore indicates that high-risk groups may benefit from a PDAC-specific screening approach. Moreover, the number of false-negative results leads to high costs for further diagnosis and insecurities for tested individuals. However, for some cancer types, the sensitivity reached relatively high values (e.g., 100% (CancerSEEK), 100% (Galleri), and 75% (THUNDER) for diagnosing stage I liver cancer). With respect to the diagnosis of advanced stages, the sensitivity was 80% to 100% for several cancer types in all tests. Further investigations on early-stage cancers are necessary to establish a reliable multicancer test, which would greatly improve the diagnosis of cancer.

In many of the reported PDAC studies in this review, several limitations affected the results of the analyses. Only 34 of the 63 studies assessed the analyzed biomarkers in early-stage PDAC patient samples. Notably, surgery is most efficient treatment in the early stages of PDAC and can significantly improve the OS of patients [5, 174, 175]. Thus, studies focusing on samples of early-stage patients are urgently needed to find suitable biomarkers for early diagnosis. Moreover, many studies analyzing single or multiple biomarkers had small samples sizes, resulting in low statistical power. Considering the high heterogeneity of patients and tumors, large cohorts (optimally from multiple centers) are needed to reliably assess biomarkers. Another limitation of most studies is the retrospective study design. Although many samples were collected prospectively, the analysis was performed retrospectively on chosen samples. Only three biomarker panels were tested in a prospective study [115, 129, 176], yielding a higher level of medical evidence. Another important aspect for future studies is the standardization of preanalytical factors as well as methods used for detection. Multicenter evaluation of CTCs, DNAs, and miRNAs in standardized blood samples revealed significant differences between the technologies used at different centers [177–179], which prevents the comparison of results and thereby limits the development of novel diagnostic assays. Therefore, it is necessary to use standardized protocols for the handling of samples and the performance of assays to improve the quality of studies. Several international liquid biopsy consortia and societies, including the European Liquid Biopsy Society (ELBS) and the International Liquid Biopsy Standardization Alliance (ILSA), are currently collaborating on this task. The establishment of reference and uniform cutoff values for promising biomarkers is important for performing randomized prospective studies to obtain more robust medical evidence. These prospective studies must be hypothesis-driven and have defined enrollment criteria to avoid the risk of missing many specific features related to the complex clinical condition of pancreatic cancer to address unmet needs. Moreover, after these studies have been completed, it is highly important to systematically review or conduct a meta-analysis of the available studies to select the best features and guide further diagnostic assay development.

## Conclusion and perspectives

This review of studies on using multimarker panels in blood samples to diagnose PDAC revealed that the use of panels of multiple biomarkers compared with single biomarkers improved the diagnostic power. Most studies have been performed on protein panels, whereas only a few have analyzed other biomarker types or even multiomic panels. Although many biomarkers had low diagnostic power alone, the combination of these biomarkers with CA19-9 increased the diagnostic power; thus, CA19-9 is part of many multibiomarker panels. For future studies, it is crucial to conduct prospective studies with standardized methods and to use samples of patients in the early stages to enable the development of a biomarker panel for early-stage PDAC diagnosis, allowing early therapeutic intervention. Owing to the low number of early-stage PDAC patients, collaborative efforts in a multicenter setting are needed. Currently, 361 ongoing studies on early-stage diagnosis of pancreatic cancer are listed at clinicaltrials.gov, illustrating the unmet need for reliable early detection methods. More than 40% of these ongoing studies (152 studies) include liquid biopsy-based diagnostics, and four of these liquid biopsy-based studies are MCED tests that utilize DNA methylation or multiomic panels. One of these recent liquid biopsy-based studies was initiated by the EU-funded PANCAID consortium [180]. Researchers in the PANCAID consortium have committed their research toward finding novel minimally invasive multimarker panels for early PDAC detection. Findings related to the early-stage detection of primary disease may also apply to the early diagnosis of minimal residual disease in cancer patients who have undergone treatment with curative intent after diagnosis [181, 182]. The European Consortium GUIDE.MRD is currently tackling this ambitious task for the detection of ctDNA in patients with pancreatic, colorectal, and lung cancers [183].

## Data Availability

Not applicable.

## Abbreviations

AC: Acute pancreatitis
apCAFs: Antigen-presenting CAFs
AUC: Area under the curve
BD: Benign disease
CA19-9: Carbohydrate antigen 19–9
CAF: Cancer-associated fibroblast
cCAF: Circulating CAF
cfDNA: Cell-free DNA
cfRNA: Cell-free RNA
CHIP: Clonal hematopoiesis of indeterminate potential
CP: Chronic pancreatitis
CTC: Circulating tumor cell
ctDNA: Circulating tumor DNA
DM: Diabetes mellitus
ECLIA: Electrochemiluminescent-based immunoassays
EIA: Enzyme immunoassay
ELISA: Enzyme-linked immunosorbent assay
EV: Extracellular vesicle
HC: Healthy control
IA: Immunoassay
IAR: Individuals at risk
iCAF: Inflammatory CAF
IPMN: Intraductal papillary mucinous neoplasm
LB: Liquid biopsy
MCED: Multi-cancer early detection
MCN: Mucinous cystic neoplasm
MIA: Magnetic immunoassay
miRNA: MicroRNAs
MOB: Methylation on beads
mRNA: Messenger RNA
MS: Mass spectrometry
myCAF: Myofibroblast CAF
OS: Overall survival
PanIN: Pancreatic intraepithelial neoplasm
PC: Pancreatic cyst
PCR: Polymerase chain reaction
PDAC: Pancreatic ductal adenocarcinoma
PFS: Progression-free survival
PLA: Proximity ligation assay
RIA: Radioimmunoassay
SN: Sensitivity
SP: Specificity
TME: Tumor microenvironment
